# Supplementary material for: Crystal structures of an E1–E2–ubiquitin thioester mimetic reveal molecular mechanisms of transthioesterification
Source: Nat Commun. 2021 Apr 22;12:2370. doi: 10.1038/s41467-021-22598-y (PMC8062481; doi:10.1038/s41467-021-22598-y)
Supplement: Supplementary file 1 — Supplementary Information [file 41467_2021_22598_MOESM1_ESM.pdf]

Supplementary Information

Crystal structures of an E1-E2-ubiquitin thioester mimetic reveal molecular mechanisms of transthioesterification

Lingmin Yuan[1,2], Zongyang Lv[1,2], Melanie J. Adams[1], & Shaun K. Olsen[1,2*]

[1]Department of Biochemistry & Molecular Biology and Hollings Cancer Center, Medical University of South Carolina, Charleston, SC, 29425, USA
[2]Department of Biochemistry & Structural Biology University of Texas Health Science Center at San Antonio, San Antonio, TX, 78229 USA

* Correspondence should be addressed to S.K.O. (olsens@uthscsa.edu)

Keywords: E1, E2, E3, ubiquitin, cell cycle, conformational change

Supplementary Information Inventory:

Supplementary Table 1-3
Supplementary Figures 1-9

Supplementary Table 1 | Crystallographic Data and Refinement Statistics

	Uba1-Cdc34^{A141K}-Ub(t) complex
PDB ID	7K5J
Source	APS 24 IDC
Wavelength (Å)	0.979
Resolution Limits (Å)	187.8-3.43 (3.62-3.43)
Space Group	$P2_1$
Unit Cell (Å) *a*, *b*, *c*	95.2, 272.9, 258.3
Unit Cell (°) α, β, γ	90, 94.6, 90
Number of crystals	2
Number of observations	2359637
Number of reflections	175210 (25582)
Completeness (%)	99.8 (100)
Mean I/σI	9.0 (1.6)
$CC_{1/2}$	0.993 (0.705)
R_{merge} [a]	0.287 (2.13)
R_{pim}	0.084 (0.612)
Refinement Statistics	
Resolution Limits (Å)	136.5 -3.43 (3.51-3.43)
# of reflections (work/free)	172677/2014
Completeness (%)	99.0 (89.0)
Protein/ligand atoms	76482/161
R_{cryst} [b]	0.203 (0.328)
R_{free} (2014 reflections)	0.246 (0.378)
Bonds (Å)/ Angles (°)	0.002/0.489
B-factors: protein/ligand (Å^2)	144.2/128.5
Ramachandran plot statistics (%)	
favored	98.0
allowed	2.2
outliers	0
MolProbity score	1.24- 99th percentile (N=27675, 0 Å - 99 Å)

Parentheses indicate statistics for the high-resolution data bin for x-ray data.
a. $R_{merge} = \sum_{hkl} \sum_i |I(hkl)_i - \langle I(hkl)\rangle| / \sum_{hkl} \sum_i \langle I(hkl)_i \rangle$.
b. $R_{cryst} = \sum_{hkl} |Fo(hkl) - Fc(hkl)| / \sum_{hkl} |Fo(hkl)|$, where *Fo* and *Fc* are observed and calculated structure factors, respectively.

Supplementary Table 2 | Ordered amino acids in the Uba1-Cdc34^{A141K}-Ub(t) structure by chain

Protein	Chain	Ordered Amino Acids
Uba1	A	12-592, 599-646, 653-745, 752-773 and 798-1024
	C	12-591, 599-645, 650-745, 752-773 and 797-1024
	D	12-590, 600-646, 650-745, 752-773 and 797-1024
	G	12-591, 600-646, 650-745, 752-773 and 797-1024
	K	12-589, 598-646, 650-773 and 797-1024
	I	12-590, 599-646, 650-773 and 797-1024
	S	12-589, 599-646, 650-773 and 797-1024
	U	12-590, 599-646, 650-745, 752-773 and 797-1024
Cdc34	B	6-95 and 117-179
	E	7-94 and 117-179
	F	7-95 and 117-179
	H	7-96 and 118-179
	L	6-98 and 118-179
	J	6-98 and 118-180
	T	6-97 and 118-178
	V	7-89 and 118-180
Ub	M	1-76
	N	1-76
	O	1-73
	P	1-76
	Q	1-76
	R	1-73
	W	1-70
	X	1-70

Supplementary Table 3 | All PCR primers used in these studies

ScUba1 C600A	Mutated from ScUba1_WT
C600A_F	gaaaagtctatcccattggctaccctacgttctttccca
C600A_R	tgggaaagaacgtagggtagccaatgggatagacttttc
ScUba1 D188A	Mutated from ScUba1_WT
D188A_F	cactggtatggtttcagccatcgagcccgatgga
D188A_R	tccatcgggctcgatggctgaaaccataccagtg
ScUba1 E190A	Mutated from ScUba1_WT
E190A_F	gtatggtttcagacatcgcgcccgatggaacagtg
E190A_R	cactgttccatcgggcgcgatgtctgaaaccatac
ScUba1 T196A	Mutated from ScUba1_WT
T196A_F	catcgagcccgatggagcagtgaccatgctagatg
T196A_R	catctagcatggtcactgctccatcgggctcgatg
ScUba1 F236A	Mutated from ScUba1_WT
F236A_F	gaggttttggggcccgctgcattcagaattggttc
F236A_R	gaaccaattctgaatgcagcgggccccaaaacctc
ScUba1 E705A	Mutated from ScUba1_WT
E705A_F	caagacttccaacggtgcaccattttggtccggt
E705A_R	accggaccaaaatggtgcaccgttggaagtcttg
ScUba1 N703A	Mutated from ScUba1_WT
N703A_F	ggatgccaagacttccgccggtgaaccattttggt
N703A_R	accaaaatggttcaccggcggaagtcttggcatcc
ScUba1 T601V	Mutated from ScUba1_WT
T601V_F	gtctatcccattgtgtgtcctacgttctttcccaaaca
T601V_R	tgtttgggaaagaacgtaggacacacaatgggatagac
ScUba1 D188L/E190L	Mutated from ScUba1_WT
D188L/E190L_F	gaacctcgcactggtatggtttcactgatcctgcccgatggaacagtgaccatgct
D188L/E190L_R	agcatggtcactgttccatcgggcaggatcagtgaaaccataccagtgcgaggttc

ScUba1 D188L/T196V	Mutated from ScUba1_WT
D188L_F	cactggtatggtttcactgatcgagcccgatgga
D188L_R	tccatcgggctcgatcagtgaaaccataccagtg
T196V_F	cccgatggaacagtggtgatgctagatgataacaga
T196V_R	tctgttatcatctagcatcaccactgttccatcggg
ScUba1 E190L/T196V	Mutated from ScUba1_WT
E190L_F	ggtatggtttcagacatcctgcccgatggaacagt
E190L_R	actgttccatcgggcaggatgtctgaaaccatacc
T196V_F	cccgatggaacagtggtgatgctagatgataacaga
T196V_R	tctgttatcatctagcatcaccactgttccatcggg
ScUba1 D188L/E190L/T196V	Mutated from ScUba1_D188L/E190L
D188L/E190L/T196V_F	ctgatcctgcccgatggaacagtggtgatgctagatgataacagacacgggttgga
D188L/E190L/T196V_R	tccaacccgtgtctgttatcatctagcatcaccactgttccatcgggcaggatcag
ScUba1 D188L/E190L/T196V/L198R	Mutated from ScUba1_D188L/E190L/T196V
D188L/E190L/T196V/L198V_F	gatggaacagtggtgatgcgtgatgataacagacacgggt
D188L/E190L/T196V/L198V_R	gatggaacagtggtgatgcgtgatgataacagacacgggt
ScUba1 N703D/E705K	Mutated from ScUba1_WT
N703D/E705K_F	cttccccaaggatgccaagacttccgacggtaaaccattttggtccggtgctaagcgt
N703D/E705K_R	acgcttagcaccggaccaaaatggtttaccgtcggaagtcttggcatccttggggaag
ScUba1 T601V/N703D/E705K	Mutated from ScUba1_T601V
N703D/E705K_F	cttccccaaggatgccaagacttccgacggtaaaccattttggtccggtgctaagcgt
N703D/E705K_R	acgcttagcaccggaccaaaatggtttaccgtcggaagtcttggcatccttggggaag
ScUba1D544A	Mutated from ScUbc3_WT
D544A_F	ggattttgtcaccaacgctctagccaatgtcgacgcaagaaca
D544A_R	tgttcttgcgtcgacattggctagagcgttggtgacaaaatcc
ScUbc3 A141K	Mutated from ScUbc3_WT
A141K_F	caatatcaactcgccaaaaaatgtcgatgccgctgt
A141K_R	acagcggcatcgacattttttggcgagttgatattg
ScUbc3 N50A	Mutated from ScUbc3_WT
N50A_F	ggtgttatggtgctagctgaggattccatttatca
N50A_R	tgataaatggaatcctcagctagcaccataacacc

<u>ScUbc3 E122A</u>	Mutated from ScUbc3_WT
E122A_F	cccgtgcagaccgtggcaagtgtgttgatctct
E122A_R	agagatcaacacacttgccacggtctgcacggg
<u>ScUbc3 S123A</u>	Mutated from ScUbc3_WT
S123A_F	ccgtgcagaccgtggaagctgtgttgatctctatagt
S123A_R	actatagagatcaacacagcttccacggtctgcacgg
<u>ScUbc3 V118A</u>	Mutated from ScUbc3_WT
V118A_F	gaaacgtggtcccccgcgcagaccgtggaaagt
V118A_R	actttccacggtctgcgcgggggaccacgtttc
<u>ScUbc3 I126A</u>	Mutated from ScUbc3_WT
I126A_F	cccgtgcagaccgtggcaagtgtgttgatctct
I126A_R	agagatcaacacacttgccacggtctgcacggg
<u>ScUbc3 E133A</u>	Mutated from ScUbc3_WT
E133A_F	ctatagtatctctattagcggaccccaatatcaact
E133A_R	agttgatattggggtccgctaatagagatactatag
<u>ScUbc3 T120A</u>	Mutated from ScUbc3_WT
T120A_F	gtggtcccccgtgcaggccgtggaaagtgtgttgatct
T120A_R	agatcaacacactttccacggcctgcacgggggaccac
<u>ScUbc3 N136A</u>	Mutated from ScUbc3_WT
N136A_F	ctattagaggaccccgctatcaactcgccagca
N136A_R	tgctggcgagttgatagcggggtcctctaatag
<u>ScUbc3 S139A</u>	Mutated from ScUbc3_WT
S139A_F	gaccccaatatcaacgcgccagcaaatgtcgat
S139A_R	atcgacatttgctggcgcgttgatattggggtc
<u>ScUbc3 Q119A</u>	Mutated from ScUbc3_WT
Q119A_F	cgtggtcccccgtggcgaccgtggaaagtgtgt
Q119A_R	acacactttccacggtcgccacgggggaccacg
<u>ScUbc3 D134A</u>	Mutated from ScUbc3_WT
D134A_F	gtatctctattagaggcccccaatatcaactcgcca
D134A_R	tggcgagttgatattgggggcctctaatagagatac

ScUbc3 L131A	gatctctatagtatctgcattagaggaccccaatatca
L131A_F	tgatattggggtcctctaatgcagatactatagagatc
L131A_R	
ScUbc3 N87A	Mutated from ScUbc3_WT
N87A_F	gctatctaccatccagccgtttacagggatggca
N87A_R	tgccatccctgtaaacggctggatggtagatagc

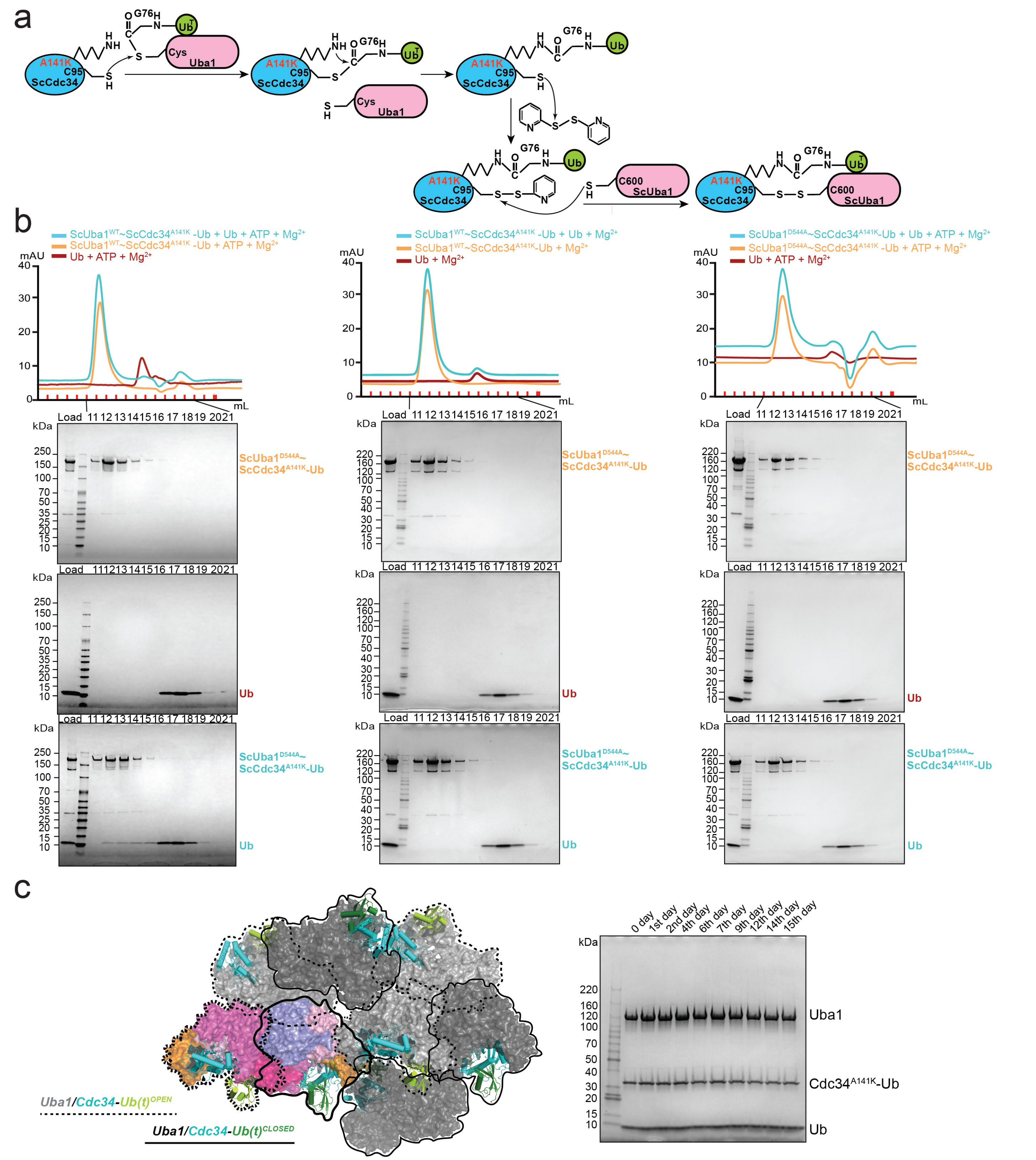

Supplementary Figure 1 | Free Ub is adenylated in the Uba1/Cdc34-Ub(t) complex in solution with ATP•Mg^{2+}

a strategy for producing E1/E2-Ub(t) mimetic. **b** *Left*, Free Ub is adenylated in the $Uba1^{WT}$/Cdc34-Ub(t) crosslinking complex in the presence of ATP and Mg^{2+}. *Middle*, Free Ub can not be adenylated in the $Uba1^{WT}$/Cdc34-Ub(t) crosslinking complex without ATP. *Right*, Free Ub can not be adenylated in the adenylation deficient mutant $Uba1^{D544A}$/Cdc34-Ub(t) crosslinking complex even in the presence of ATP and Mg^{2+}. **c** *Left*, The Uba1/$Cdc34^{A141K}$-Ub(t) structure contains eight copies in the crystallographic asymmetric unit where four Ub(t) s adopt open state and the other four adopt closed state arrangements. *Right*, SDS-PAGE gel of Uba1/Cdc34-Ub(t)/Ub(a)/ATP•Mg^{2+} crystallization sample from different days under reducing loading buffer. All gel images are representative of independent technical replicates (*n* = 2). Source data are provided as a Source Data file.

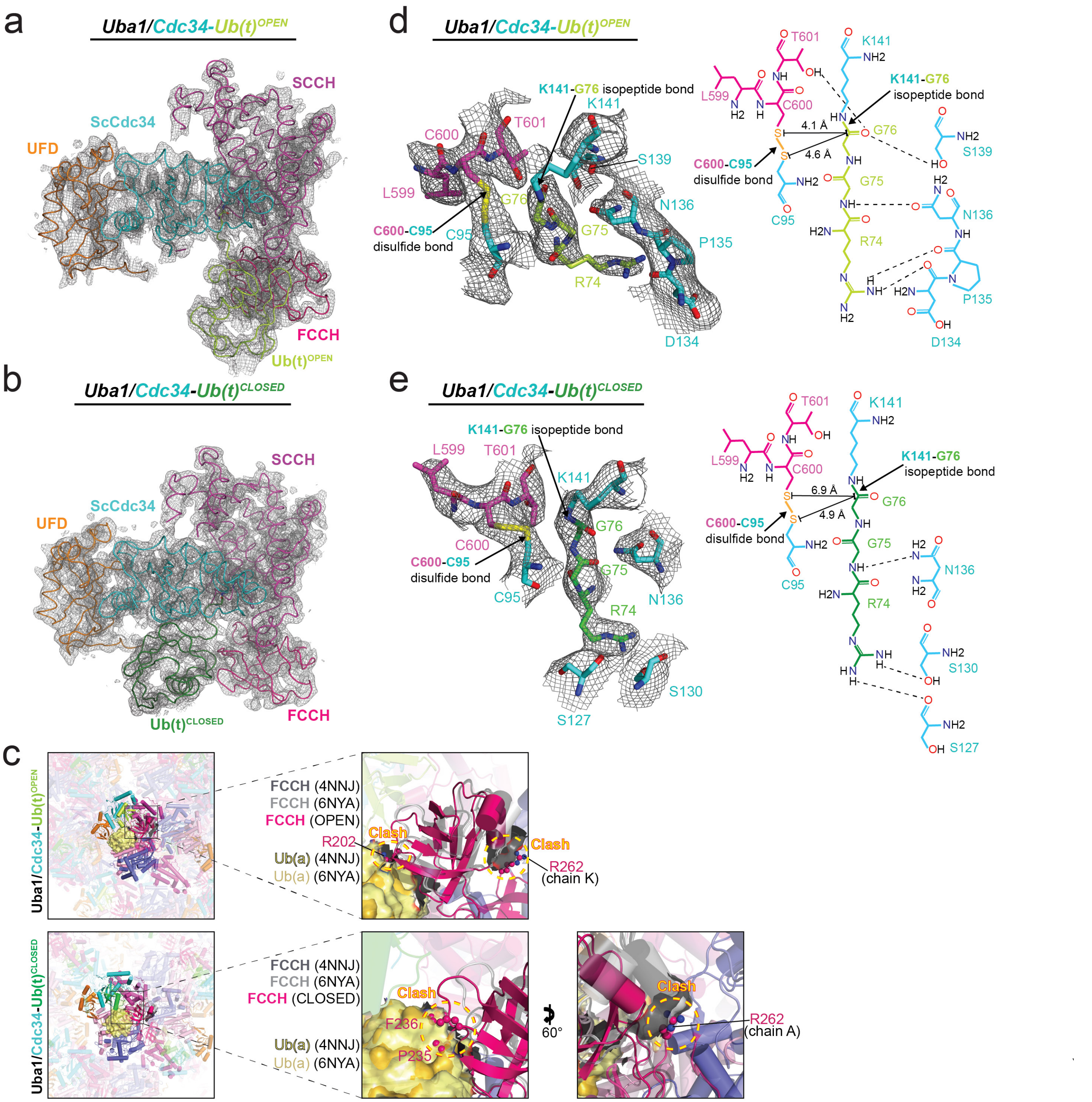

Supplementary Figure 2 | Density maps of Cdc34-Ub(t) complex with Uba1

a 2Fo-Fc electron density maps contoured at 1.0σ for Uba1/Cdc34-Ub(t)^{OPEN} overall structure with Cα-trace shown. **b** 2Fo-Fc electron density maps contoured at 1.0σ for Uba1/Cdc34-Ub(t)^{CLOSED} overall structure with Cα-trace shown. **c** *Top*, Symmetry mates within 10 Å of the Uba1/Cdc34-Ub(t)^{OPEN} structure. Uba1 domains are colored as in Fig. 2: IAD is in slate, AAD is in pink, FCCH is in hotpink, SCCH is in light magenta, UFD is in orange. Cdc34^{A141K} is colored cyan, and Ub(t)^{OPEN} is colored limon. Ub(a)s from ScUba1/Ub(t)/Ub(a) (PDB: 4NNJ) and Uba1/Cdc34/Ub(a) (PDB: 6NYA) are docked into Uba1/Cdc34-Ub(t)^{OPEN} structure by superposition of Uba1 AD domains. Ub(a) (PDB: 4NNJ), ScUba1 FCCH (PDB: 4NNJ), Ub(a) (PDB: 6NYA), ScUba1 FCCH (PDB: 6NYA) are colored and labeled. Ub(a) (PDB: 4NNJ) and Ub(a) (PDB: 6NYA) are shown as surface representation. Arg202 of FCCH (OPEN) would clash with the docked Ub(a)s, but crystal packing from adjacent Arg262 (chain K) perturbed FCCH(OPEN) orientation to accommodate Ub(a)s and consequently occludes Ub(a) from occupying the AAD binding site. *Bottom*, Symmetry mates within 10 Å of the Uba1/ Cdc34-Ub(t)^{CLOSED} structure. Uba1 domains are colored as in Fig. 2: IAD is in slate, AAD is in pink, FCCH is in hotpink, SCCH is in light magenta, UFD is in orange. Cdc34^{A141K} is colored cyan, and Ub(t)^{CLOSED} is colored forest. Ub(a)s from ScUba1/Ub(t)/Ub(a) (PDB: 4NNJ) and Uba1/Cdc34/Ub(a) (PDB: 6NYA) are docked into Uba1/Cdc34-Ub(t)^{CLOSED} structure by superposition of Uba1 AD domains. Ub(a) (PDB: 4NNJ), ScUba1 FCCH (PDB: 4NNJ), Ub(a) (PDB: 6NYA), ScUba1 FCCH (PDB: 6NYA) are colored and labeled. Ub(a) (PDB: 4NNJ) and Ub(a) (PDB: 6NYA) are shown as surface representation. Pro235 and Phe236 of FCCH (CLOSED) would clash with the docked Ub(a)s, but crystal packing from adjacent Arg262 (chain A) perturbed FCCH(CLOSED) orientation to accommodate Ub(a)s and consequently occludes Ub(a) from occupying the AAD binding site. **d** *Left*, 2Fo-Fc electron density maps contoured at 1.0σ for E1, E2, Ub(t) active sites in Uba1/Cdc34-Ub(t)^{OPEN} structure. *Right*, Chemical drawing as shown in **d** *Left*. **e** *Left*, 2Fo-Fc electron density maps contoured at 1.0σ for E1, E2, Ub(t) active sites Uba1/Cdc34-Ub(t)^{CLOSED} structure. *Right*, Chemical drawing as shown in **e** *Left*.

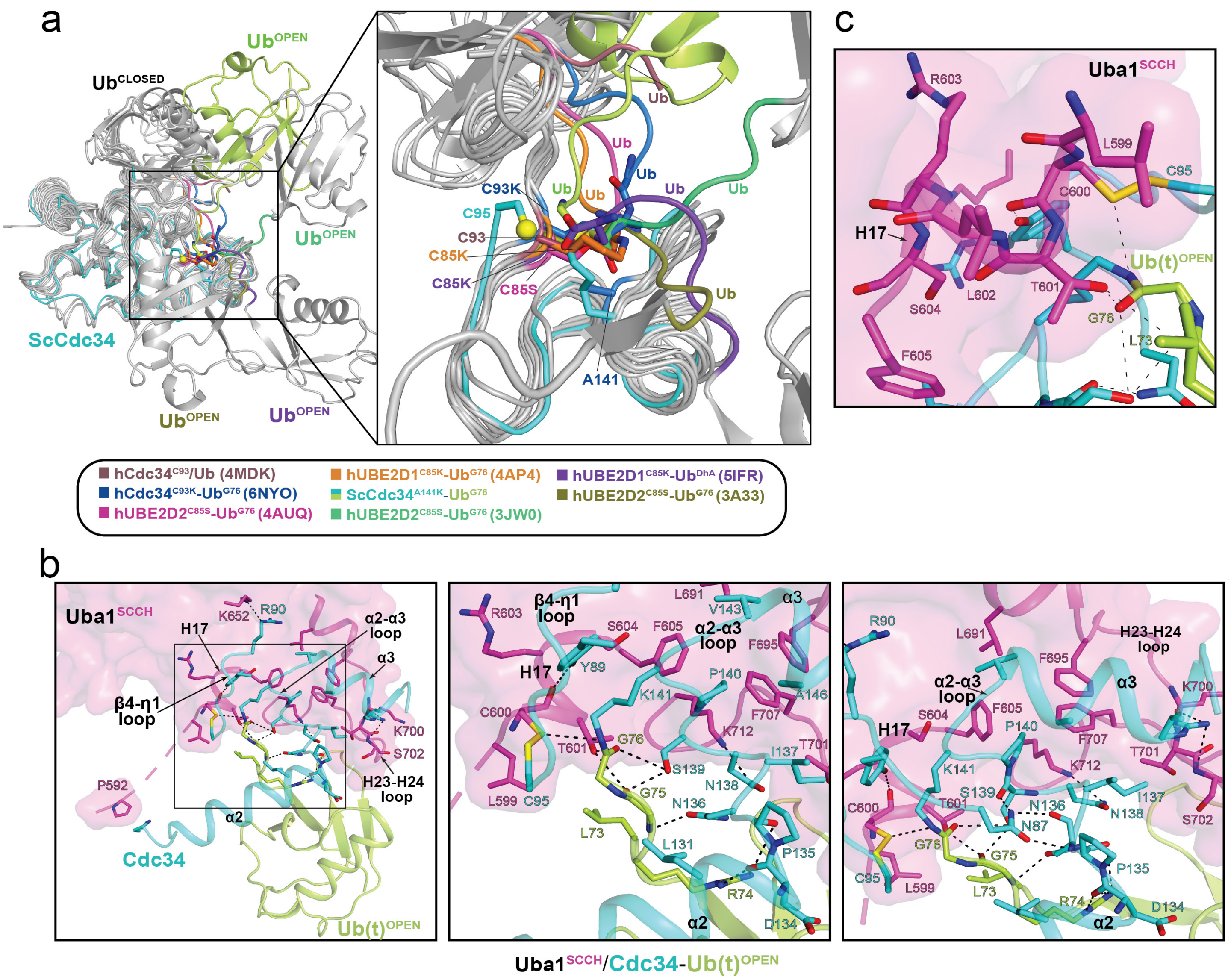

Supplementary Figure 3 | Structure analysis for Uba1/Cdc34-Ub(t)^{OPEN}

a Comparison of E2-Ub open and closed structures by E2 superposition. **b** Interfaces among Uba1 SCCH domain, Cdc34 and Ub(t)^{OPEN}. **c** N-Terminal of Uba1 H17 is in proximity to the Ub(t)^{OPEN} Gly76 carbonyl oxygen where the positive electrostatic potential from the helix dipole could contribute to stabilization of the transition state.

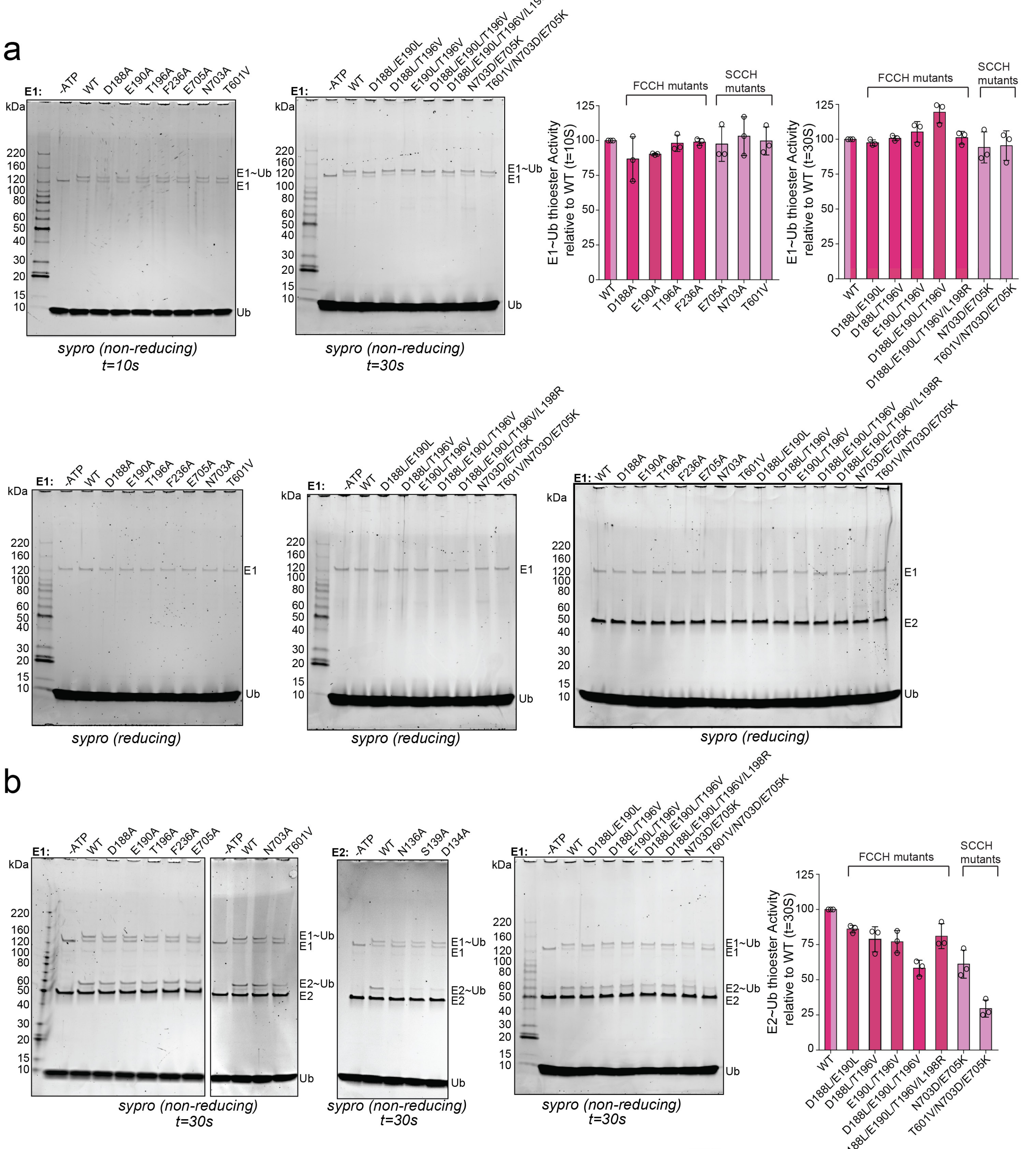

Supplementary Figure 4 | Structure function analysis for Uba1/Cdc34-Ub(t)^{OPEN}

a *Top*, E1~Ub thioester assay and quantification for the indicated ScUba1 residues involved in Uba1/Ub(t) interface. Data are represented by mean ± SD with three independent technical replicates labeled above and individual replicates shown as black circles. Gel images are representative of independent technical replicates (*n* = 3). *Bottom*, E1~Ub thioester assay and E2~Ub thioester assay for the indicated ScUba1 residues involved in Uba1/Ub(t) interface in presence of DTT. Gel images are representative of independent technical replicates (*n* = 2). **b** *Left* and *Middle*, Full gels for E1-E2 thioester transfer assay in Fig. 3. Gel images are representative of independent technical replicates (*n* = 3). *Right*, E1~Ub thioester assay and quantification for the indicated ScUba1 residues involved in Uba1/Ub(t) interface. Data are represented by mean ± SD with three independent technical replicates labeled above and individual replicates shown as black circles. Gel image is representative of independent technical replicates (*n* = 3). Source data for all panels in this figure are provided as a Source Data file.

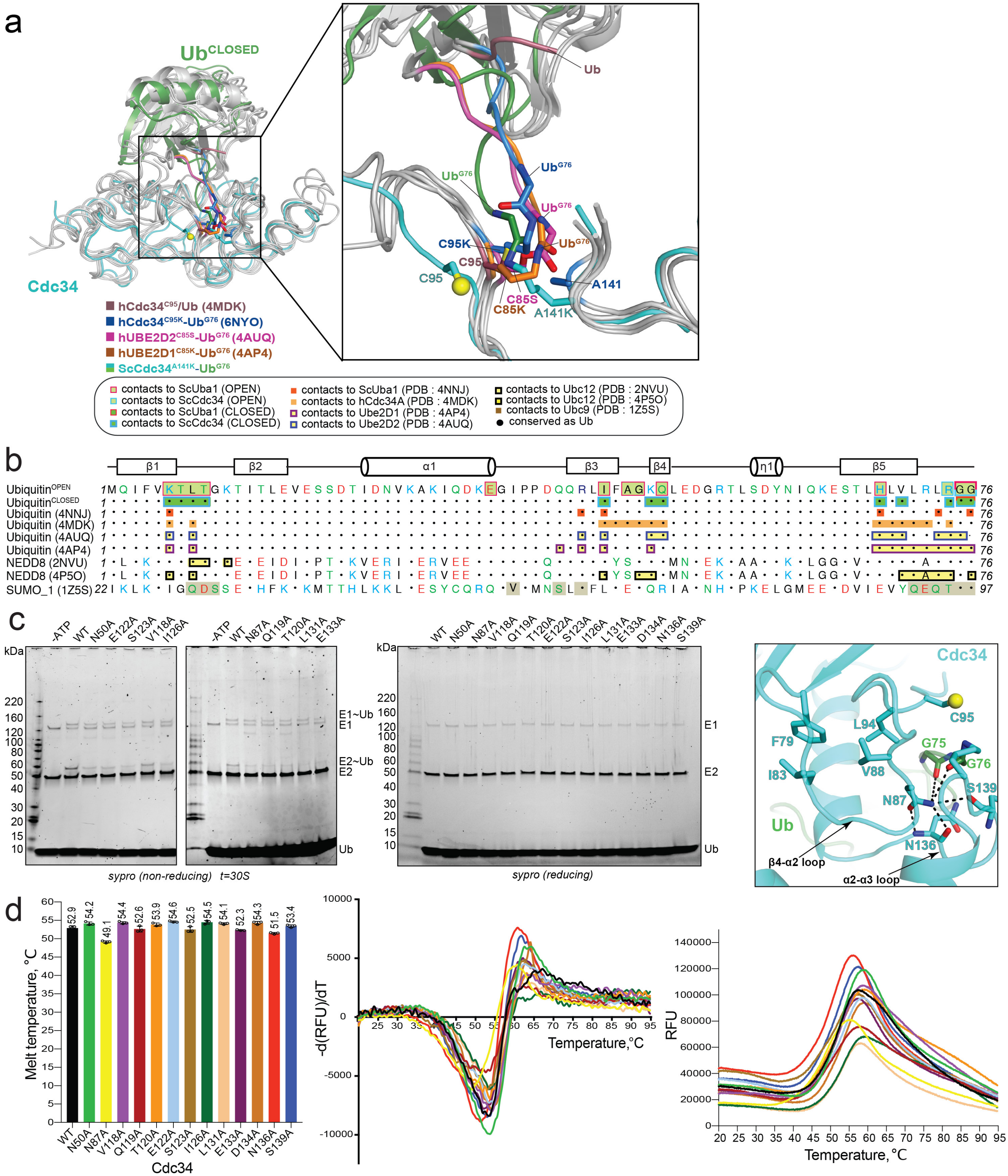

Supplementary Figure 5 | Structure function analysis for Uba1/Cdc34-Ub(t)^{CLOSED}

a Comparison of E2-Ub closed structures by E2 superposition. **b** Sequence alignment of Ub, Nedd8 and SUMO_1 with Ub's secondary structure cartoon shown above sequence. Shaded boxes in Ub(t)^{OPEN} indicate residues that interact with Uba1, and others indicate residues of Ub, Nedd8 or SUMO_1 that interact with E2s. **c** *Left,* Full gels for E1-E2 thioester transfer assay in Fig. 4. Gel images are representative of independent technical replicates (*n* = 3). *Middle*, Full gel for E1-E2 thioester transfer assay in presence of DTT. Gel image is representative of independent technical replicates (*n* = 2). *Right*, Cdc34 Asn87 involves in intramolecular interaction. **d** Thermal shift assay of Cdc34 WT and mutants for E1-E2 thoester transfer in Fig. 3 and Fig. 4. Data are represented by mean ± SD with three independent technical replicates labeled above and individual replicates shown as black circles. Source data for all panels in this figure are provided as a Source Data file.

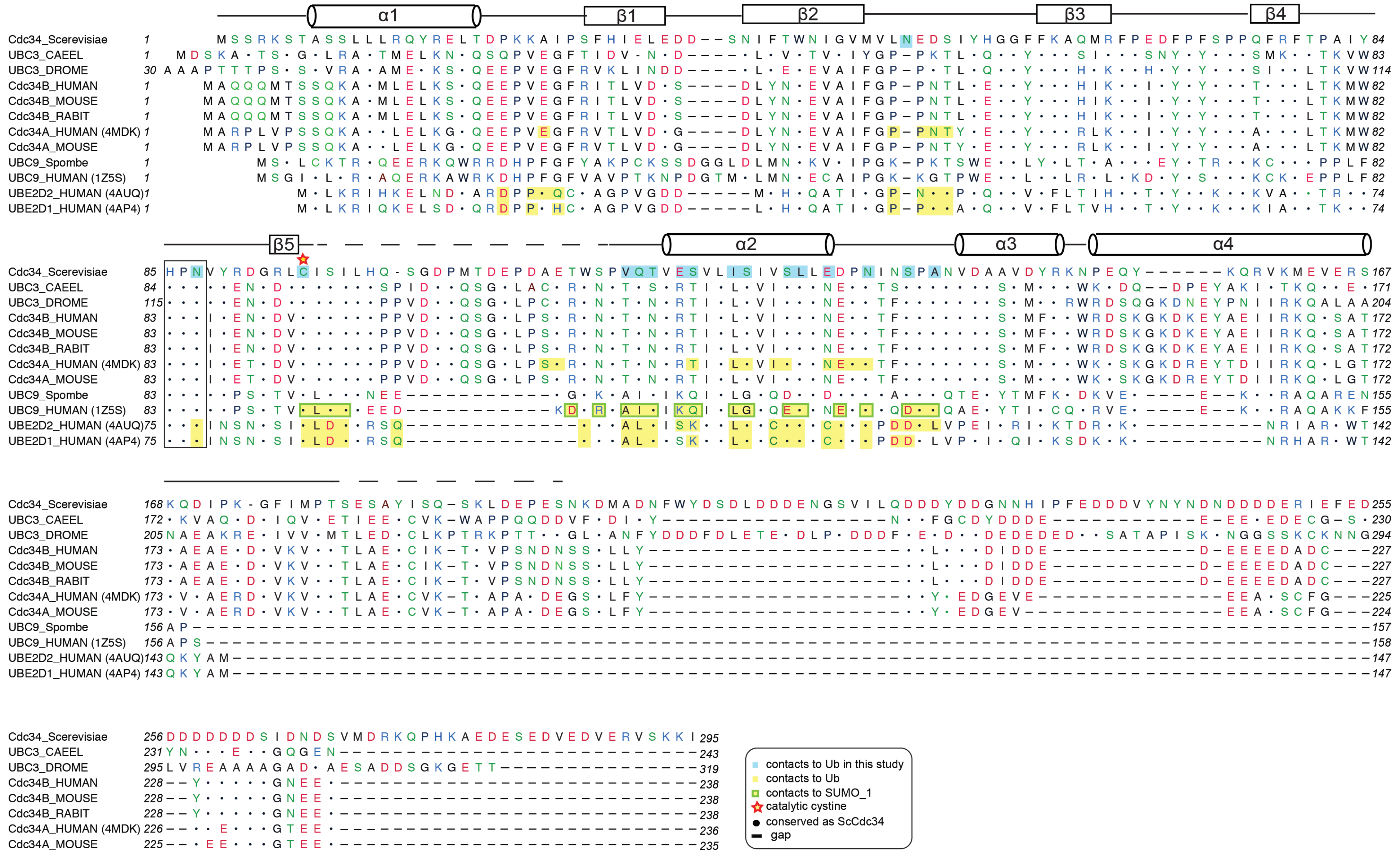

Supplementary Figure 6 | Sequence alignment of Cdc34 across different species

Secondary structure of ScCdc34 in our study is represented above the aligned sequence. Shaded boxed residues of E2s indicate contacts on UBLs. HPN motif is boxed. A region of disorder in the ScCdc34 structure is indicated as a dashed line.

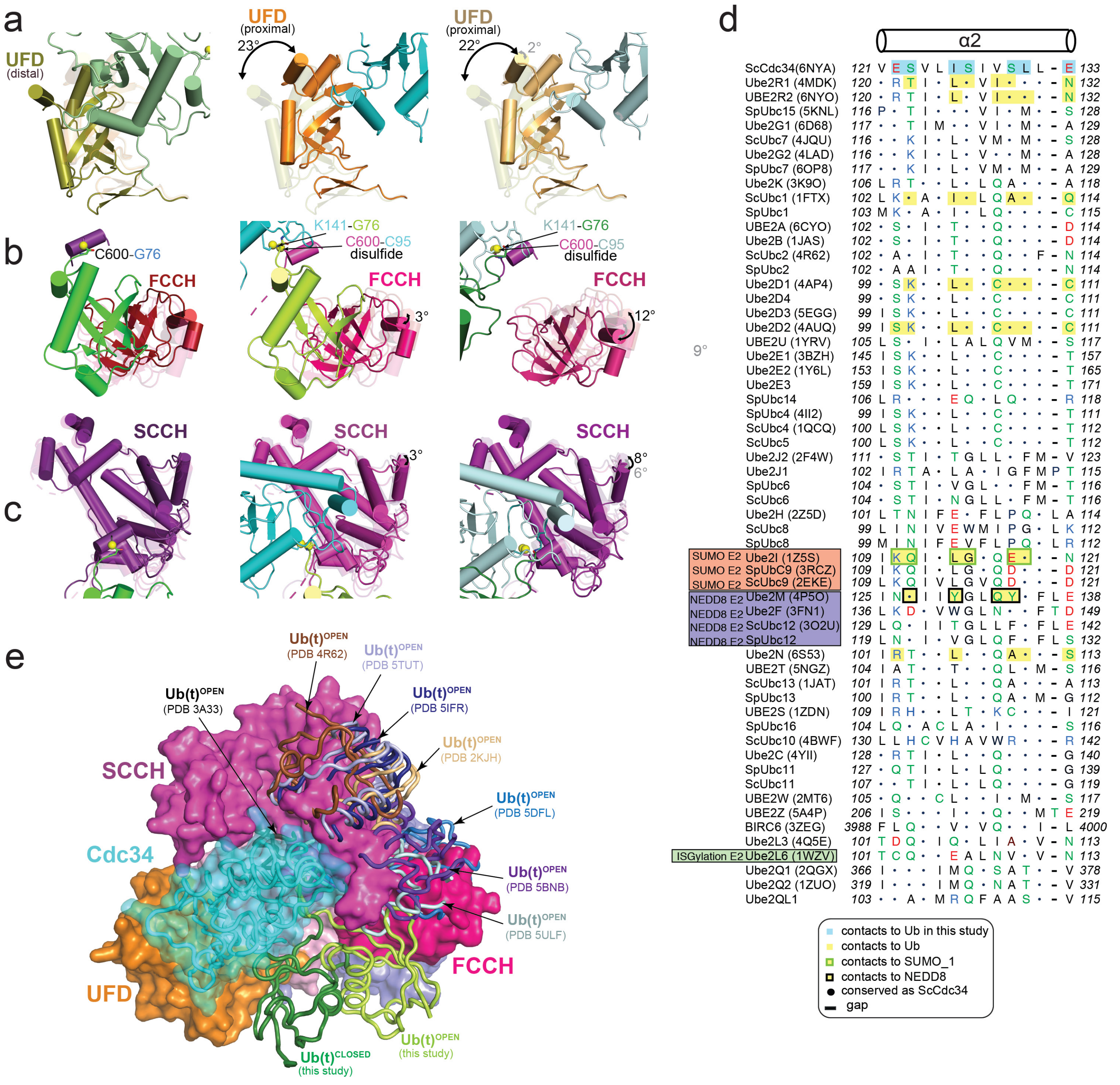

Supplementary Figure 7 | Comparison of docking model, Uba1/Cdc34^{OPEN} and Uba1/Cdc34^{CLOSED} structures

a UFD domain rotates ~23º after Cdc34 binding for E1 E2 catalytic sites coming into proximity. With regard to the docking model, the domain rotation is marked black, while with regard to the open structure, the domain rotation of closed structure is marked gray. Docking model (*Left*),open structure (*Middle*), and closed structure (*Right*) represented as in Fig. 5. **b** FCCH domain comparison among these three structures. With regard to the docking model, the domain rotation is marked black, while with regard to the open structure, the domain rotation of closed structure is marked gray. Docking model (*Left*), open structure (*Middle*), and closed structure (*Right*) represented as in Fig. 5. **c** SCCH domain comparison among these three structures. With regard to the docking model, the domain rotation is marked black, while with regard to the open structure, the domain rotation of closed structure is marked gray. Docking model (*Left*), open structure (*Middle*), and closed structure (*Right*) represented as in Fig. 5. **d** Structure-based sequence alignment for E2s' helix α2 depicting regions contacting Ub^{CLOSED} or Ubl^{CLOSED} . **e** Model of Ub(t)^{OPEN} positions on Uba1 derived by superposition of E2s from available E2~Ub^{OPEN} thioester structures on to Cdc34 in the Uba1/Cdc34^{CLOSED} structure. Uba1 and Cdc34 are in surface representation with domains colored as in Figure 2.

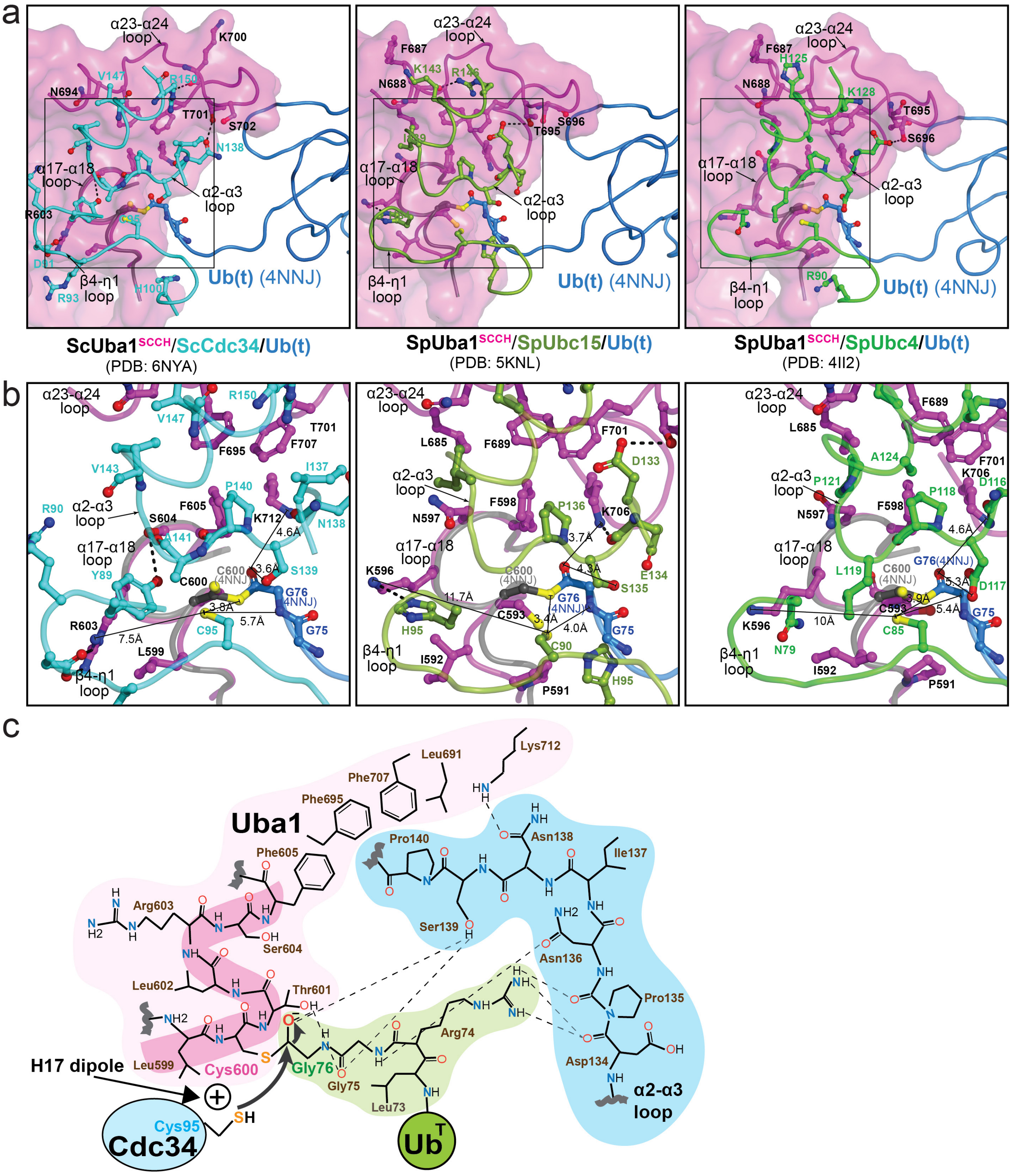

Supplementary Figure 8 | Ub E1-E2 thioester transfer catalytic mechanism

a Ub(t) (PDB : 4NNJ) is docked into E1-E2 crosslinking structures ScUba1/ScCdc34 (PDB : 6NYA), SpUba1/SpUbc15 (PDB : 5KNL), and SpUba1/SpUbc4 (PDB : 4II2) with superposition of SCCH domain. SCCH domain is surface represented and colored magenta. **b** Zoom in of E1, E2 and Ub(t) catalytic sites, *Left*, ScUba1/ScCdc34/Ub(t) model, *Middle*, SpUba1/SpUbc15/Ub(t) model, *Right*, SpUba1/SpUbc4/Ub(t) model as in **a**. **c** Model for catalysis of Ub E1-E2 thioester transfer formation by using *S. cerevisiae* Uba1/Cdc34/Ub(t) as template.

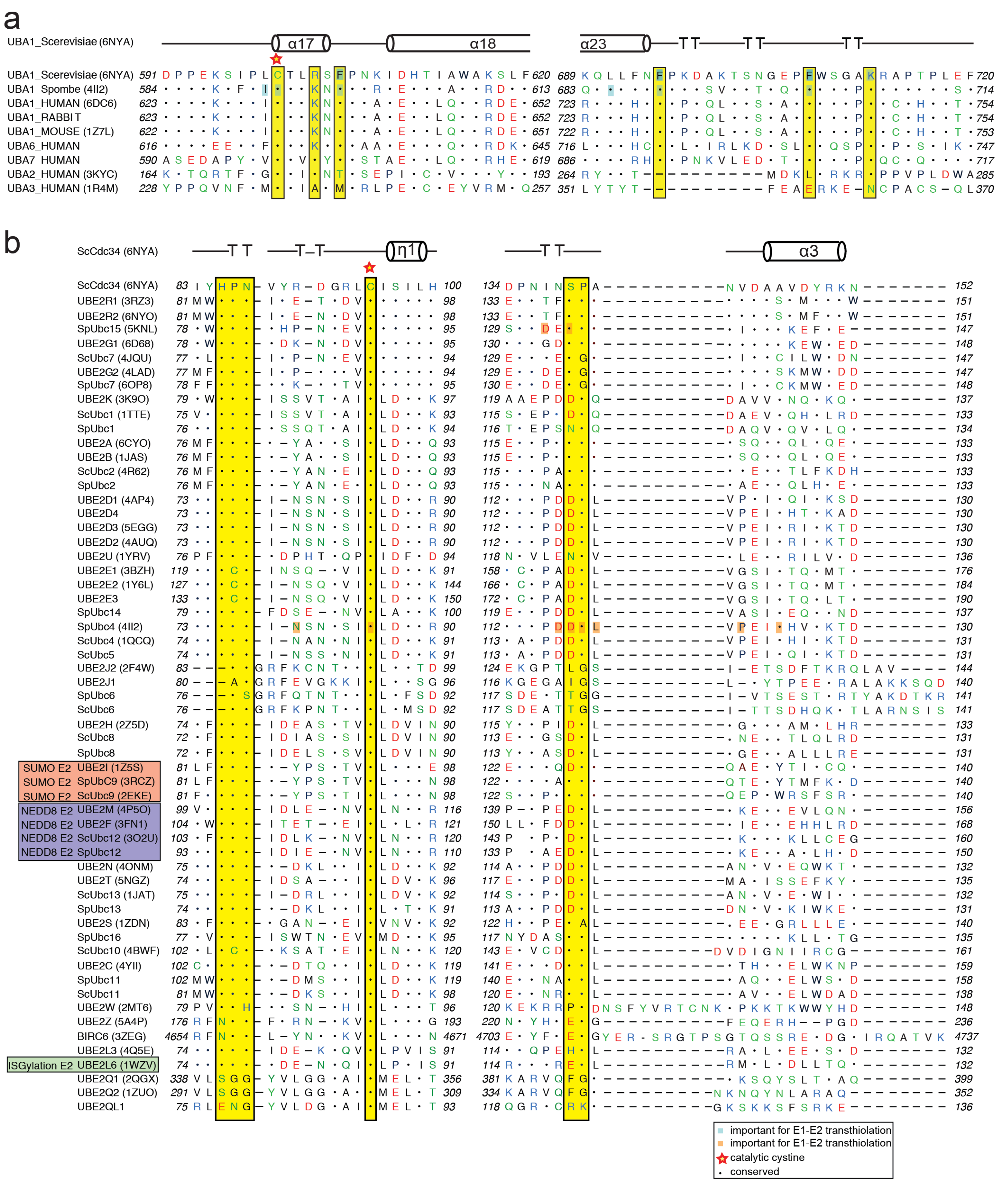

Supplementary Figure 9 | Structure based sequence alignment of E1s and E2s.
a Sequence alignment of Uba1 across species and Uba6, ISGylation E1 Uba7 and Sumo E1 Uba 2 and Nedd8 E1 Uba3. Secondary structure of ScUba1 (6NYA) is represented above the aligned sequence. Shaded boxed residues indicate residues important for thioester transfer. Residues related to E1-E2catalysis are boxed. **b** Sequence alignment of *S. cerevisiae* E2s, *S.pombe* E2s and activated human E2s. Secondary structure of ScCdc34 (6NYA) is represented above the aligned sequence.Shaded boxed residues indicate residues important for thioester transfer. Residue related to E1-E2 catalysis are boxed. HPN motif is boxed.
