# Supplementary material for: Crystal structures of an E1–E2–ubiquitin thioester mimetic reveal molecular mechanisms of transthioesterification
Source: Nat Commun. 2021 Apr 22;12:2370. doi: 10.1038/s41467-021-22598-y (PMC8062481; doi:10.1038/s41467-021-22598-y)
Supplement: Supplementary file 3 — Reporting Summary [file 41467_2021_22598_MOESM3_ESM.pdf]

nature research

Corresponding author(s): Shaun K. Olsen

Last updated by author(s): 2021/03/02

Reporting Summary

Nature Research wishes to improve the reproducibility of the work that we publish. This form provides structure for consistency and transparency in reporting. For further information on Nature Research policies, see our Editorial Policies and the Editorial Policy Checklist.

Statistics

For all statistical analyses, confirm that the following items are present in the figure legend, table legend, main text, or Methods section.

n/a	Confirmed	
☐	☒	The exact sample size (n) for each experimental group/condition, given as a discrete number and unit of measurement
☐	☒	A statement on whether measurements were taken from distinct samples or whether the same sample was measured repeatedly
☒	☐	The statistical test(s) used AND whether they are one- or two-sided<br>*Only common tests should be described solely by name; describe more complex techniques in the Methods section.*
☒	☐	A description of all covariates tested
☒	☐	A description of any assumptions or corrections, such as tests of normality and adjustment for multiple comparisons
☐	☒	A full description of the statistical parameters including central tendency (e.g. means) or other basic estimates (e.g. regression coefficient) AND variation (e.g. standard deviation) or associated estimates of uncertainty (e.g. confidence intervals)
☒	☐	For null hypothesis testing, the test statistic (e.g. F, t, r) with confidence intervals, effect sizes, degrees of freedom and P value noted<br>*Give P values as exact values whenever suitable.*
☒	☐	For Bayesian analysis, information on the choice of priors and Markov chain Monte Carlo settings
☒	☐	For hierarchical and complex designs, identification of the appropriate level for tests and full reporting of outcomes
☒	☐	Estimates of effect sizes (e.g. Cohen's d, Pearson's r), indicating how they were calculated

Our web collection on statistics for biologists contains articles on many of the points above.

Software and code

Policy information about availability of computer code

Data collection	Crystallography data collection was conducted remotely using the in-house software of either NE-CAT or SER-CAT at the Advanced Photon Source.
Data analysis	Crystallography data analysis was conducted using the HKL2000 v717, PHENIX v1.12, and COOT 0.91 software suites. Ubiquitin thioester activity data analysis was conducted using ImageJ 1.53 and GraphPad Prism 7.0a. Thermal shift data was analysied by GraphPad Prism 7.0a.

For manuscripts utilizing custom algorithms or software that are central to the research but not yet described in published literature, software must be made available to editors and reviewers. We strongly encourage code deposition in a community repository (e.g. GitHub). See the Nature Research guidelines for submitting code & software for further information.

Data

Policy information about availability of data

All manuscripts must include a data availability statement. This statement should provide the following information, where applicable:

- Accession codes, unique identifiers, or web links for publicly available datasets
- A list of figures that have associated raw data
- A description of any restrictions on data availability

Atomic coordinates and structure factors are deposited in the RCSB with accession code 7K5J. Coordinates and structure factors for protein structures discussed or used in analyses are accessible at the RCSB. The structural data used from RCSB are listed below: PDB: 4NNJ, PDB: 6NYA, PDB: 2NVU, PDB: 3CMM, PDB: 6NYD, PDB: 4MDK, PDB: 1Z5S, PDB: 4AUC, PDB: 4AP4, PDB: 4P5O, PDB: 4II3, PDB: 6O83, PDB: 5IFR, PDB: 3A33, PDB: 3JW0, PDB: 5KNL, PDB: 6D68, PDB: 4JQU, PDB: 4LAD, PDB: 6OP8, PDB: 3K9O, PDB: 1FTX, PDB: 6CYO, PDB: 1JAS, PDB: 4R62, PDB: 4R62, PDB: 5EGG, PDB: 1YRV, PDB: 3BZH, PDB: 1Y6L, PDB: 4II2, PDB: 1QCQ, PDB: 2F4W, PDB: 2Z5D, PDB: 3RCZ, PDB: 2EKE, PDB: 3FN1, PDB: 3O2U, PDB: 6S53, PDB: 5NGZ, PDB: 1JAT, PDB: 1ZDN, PDB: 4BWF, PDB: 4YII, PDB: 2MT6, PDB: 5A4P, PDB: 3ZEG, PDB: 4Q5E, PDB: 1WZV, PDB: 2QGX, PDB: 1ZUO, PDB: 5TUT, PDB: 2KJH, PDB: 5DFL, PDB: 5BNB, PDB, 5ULF, PDB: 6DC6, PDB: 1Z7L, PDB: 3KYC, PDB: 1R4M, PDB: 3RZ3,

PDB: 1TTE, PDB: 4ONM.

The source data underlying Fig. 1b, Fig. 3c, Fig. 4c, Supplementary Figs 1b, c, 4 and 5c, d are provided as a Source Data file; any additional data may be requested from the corresponding author.

Field-specific reporting

Please select the one below that is the best fit for your research. If you are not sure, read the appropriate sections before making your selection.

☒ Life sciences ☐ Behavioural & social sciences ☐ Ecological, evolutionary & environmental sciences

For a reference copy of the document with all sections, see nature.com/documents/nr-reporting-summary-flat.pdf

Life sciences study design

All studies must disclose on these points even when the disclosure is negative.

Sample size	Due to the cleanliness and sensitivity of our biochemical assays using purified proteins, these studies have fairly robust differences in activity levels. We chose to do three independent technical replicates for each biochemical assay reported in the manuscript.
Data exclusions	No data were excluded from the analyses.
Replication	All of the biochemical data were initially produced in preliminary screening assays a total of 3 to 5 times on different days, then reproduced in publication quality replicates performed as 3 independent technical replicates at a later time. All data were reproducible across these studies.
Randomization	We did not use experimental grouping in these studies, so no randomization was necessary.
Blinding	Initial biochemical data analyses for quantification were conducted using lane and construct numbers instead of mutant identification, however, the person conducting the quantification did purify the mutants and set up the assays, so due to memory it was not a fully-blind analysis. Since the method for quantification using ImageJ was essentially objective, we do not feel that blinding was necessary, and visual confirmation of the trends is available in the manuscript by providing gel images.

Reporting for specific materials, systems and methods

We require information from authors about some types of materials, experimental systems and methods used in many studies. Here, indicate whether each material, system or method listed is relevant to your study. If you are not sure if a list item applies to your research, read the appropriate section before selecting a response.

Materials & experimental systems

n/a	Involved in the study
☒	☐ Antibodies
☒	☐ Eukaryotic cell lines
☒	☐ Palaeontology and archaeology
☒	☐ Animals and other organisms
☒	☐ Human research participants
☒	☐ Clinical data
☒	☐ Dual use research of concern

Methods

n/a	Involved in the study
☒	☐ ChIP-seq
☒	☐ Flow cytometry
☒	☐ MRI-based neuroimaging
